# The 1973 WHO Classification Is More Suitable than the 2004 WHO Classification for Predicting Prognosis in Non-Muscle-Invasive Bladder Cancer

**DOI:** 10.1371/journal.pone.0047199

**Published:** 2012-10-17

**Authors:** Zhongqing Chen, Weihong Ding, Ke Xu, Jun Tan, Chuanyu Sun, Yuancheng Gou, Shijun Tong, Guowei Xia, Zujun Fang, Qiang Ding

**Affiliations:** 1 Department of Pathology, Huashan Hospital affiliated to Fudan University, Shanghai, China; 2 Department of Urology, Huashan Hospital affiliated to Fudan University, Shanghai, China; 3 Department of Biostatistics & Social Medicine, School of Public Health, Fudan University, Shanghai, China; University of Colorado, United States of America

## Abstract

**Background:**

Predicting the recurrence and progression of Non-muscle-invasive bladder cancer(NMIBC) is critical for urologist. Histological grade provides significant prognostic information, especially for prediction of progression. Currently, the 1973 and the 2004 WHO classification co-exist. Which system is better for predicting rumor recurrence and progression still a matter for debate.

**Methodology/Principal Findings:**

348 patients diagnosed with Non-muscle invasive bladder cancer were enrolled in our retrospective study. Paraffin sections were assessed by an experienced urological pathologist according to both the 1973 and 2004 WHO classifications. Tumor recurrence and progression was followed-up in all patients. During follow-up, corresponding 5-year recurrence-free survival rates of G1, G2 and G3 were 82.1%, 55.9%, 32.1% and the 5-year progression-free survival rates were 95.9%, 84.4% and 43.3%, respectively. The 5-year recurrence-free survival rates of papillary urothelial neoplasm of low malignant potential (PUNLMP), low-grade papillary urothelial carcinoma(LGPUC) and high-grade papillary urothelial carcinoma (HGPUC) were 69.8%, 67.1% and 42.0% respectively and the 5-year progression-free survival rates were 100%, 90.9% and 54.8% respectively. In multivariate analysis, the 1973 WHO classification significantly associated with both tumor recurrence and progression(p = 0.010 and p = 0.022, respectively); the 2004 WHO classification correlated with tumor progression(p = 0.019), while was not proved to be a variable that can predict the risk of recurrence(p = 0.547). Kaplan-Meier plots showed that both the 1973 WHO and the 2004 WHO classifications were significantly associated with progression-free survival (*p*<0.0001, log-rank test). For prediction of recurrence, significant differences were observed between the tumor grades classified using the 1973 WHO grading system *(p<*0.0001, log-rank test), while a significant overlap was observed between PUNLMP and LG plots using the 2004 WHO grading system(p = 0.616, log-rank test).

**Conclusion/Significance:**

Both the 1973 WHO and the 2004 WHO Classifications are effective in predicting tumor progression in Non-muscle invasive bladder cancer, while the 1973 WHO Classification is more suitable for predicting tumor recurrence.

## Introduction

Urothelial carcinoma (UC) of the urinary bladder is the ninth most common cancer worldwide, accounting for 3% of the global cancer incidence [1]. Approximately 75–85% of patients with bladder cancer present with disease confined to the mucosa [stage Ta, carcinoma in situ (CIS)] or submucosa (stage T1) [2]. These non-muscle-invasive bladder cancer(NMIBC) shows significant patient-to-patient variability depending on disease characteristics: the probability of tumor recurrence at 1 year ranges from about 15% to 70% [3]; and the probability of tumor progression at 5 years ranges from about 7% to 40% [4]. Predicting such behavior is clinically important as invasion bears a significant risk of metastasis and impaired survival [5]–[6]. Therefore, prediction of tumor recurrence and progression is critical for determining appropriate therapy and follow-up stratification.

In 2006, the European Organization for Research and Treatment of Cancer (EORTC) Genito-Urinary Group published risk tables [7] taking into account the 6 most significant clinical and pathological factors: the number of tumors, tumor size, prior recurrence rate, T category, presence of concomitant CIS, and tumor grade. These risk tables are now widely used.

Histological grade provides significant prognostic information, especially for prediction of progression [7], [8]. The 1973 World Health Organization (WHO) classification consists of urothelial papilloma and carcinoma grades 1 to 3 [9]. In 1998, a revised grading system for urothelial carcinoma was proposed and adopted by the WHO in 2004 to replace the 1973 WHO grading system [10]. The 2004 WHO classification includes urothelial papilla, papillary urothelial neoplasm of low malignant potential (PUNLMP), low-grade urothelial carcinoma (LGPUC) and high-grade UC (HGPUC). Since then, this new system has been discussed in a number of publications. However, its value is still a matter for debate [11]–[13].

The aim of this study was to evaluate the prognostic value of both the 1973 and 2004 WHO grading systems and to verify the most suitable system for predicting tumor recurrence and progression.

## Materials and Methods

### Patient Characteristics and Ethics Statement

During the period from November 1999 to December 2009, 392 patients with bladder carcinoma who underwent transurethral resection (TUR) and had diagnosis confirmed as NMIBC by examination of paraffin-embedded blocks were assessed in Department of Urology, Huashan Hospital affiliated to Fudan University. When first seen, features of the neoplasms were detailed recorded, including tumor location, tumor size, number of tumors, and tumor shape. A total of 44 patients were excluded from the analyses: 26 lost to follow-up, 12 due to death non-related to bladder cancer and 6 due to stage downgrading (misdiagnosed T2 as T1) after histopathological review. In total, 348 eligible patients with histopathologically confirmed NMIBC were enrolled in this retrospective study based on the 2002 American Joint Committee on Cancer TNM staging system [14]. Individual patient data are shown in Table 1. Paraffin sections of tumors were obtained from the Department of Pathology, Huashan Hospital affiliated to Fudan University. The study was approved by the Institute Research Medical Ethics Committee of Fudan University School of Medicine. Data were analyzed anonymously, no informed consent was obtained for use of retrospective Paraffin sections from the patients, since this was not deemed necessary by the Ethics Committee, who waived the need for consent.

**Table 1:** Patient characteristics.

Characteristics	Value (%)
**Age(yr)**	
Median	68
Range	21–92
**Gender**	
Male	287 (82.5)
Female	61(17.5)
Tumor size(cm)	
<3 cm	233(67.0)
≥3 cm	115(33.0)
Number of tumors	
single	218 (62.6)
multiple	130 (37.4)
prior recurrence rate	
Primary	292 (83.9)
Recurrence	56 (16.1)
Carcinoma in situ	
No	327 (94.0)
Yes	21 (6.0)
Tumor stage	
Ta	220 (63.2)
T1	128 (46.8)
Tumor grade(1973)	
G1	125 (35.9)
G2	176 (50.6)
G3	47 (13.5)
Tumor grade(2004)	
PUNLMP	40(11.5)
LG	223(64.1)
HG	85(24.4)
Recurrence	
No	226 (64.9)
Yes	122 (35.1)
Progression	
No	307 (88.2)
Yes	41 (11.8)

**Table 2:** Prognostic implications of both World Health Organisation (WHO) classifications in terms of recurrence-free and progression-free survival.

	Recurrence			Progression		
	n	1 yearrecurrence-free %	5 yearrecurrence-free%	n	1 yearprogression-free%	5 yearprogression-free%
G1(n = 125)	19	97.6	82.1	3	100	95.9
G2(n = 176)	74	82.9	55.9	20	97.6	84.4
G3(n = 47)	29	59.6	32.1	18	77.9	43.4
PUNLMP(n = 40)	10	94.9	69.8	0	100	100
LG(n = 223)	67	89.7	67.1	15	99.1	90.9
HG(n = 85)	45	68.2	42.0	26	85.4	54.8

**Table 3:** Multivariate analyses of WHO 1973 classification and other clinical parameters inferences to predict tumor recurrence and progression among patients with non-muscle-invasive bladder cancer.

Variable	Recurrence		Progression		
	HR	P value	HR	P value	
Number of tumors: Single,	1.84(1.27,2.66)	0.001	1.29(0.68,2.48)	0.437	
multipleTumor Size: <3 cm, ≥3 cm	2.02(1.39,2.92)	<0.0001	2.98(1.52,5.84)	0.001	
Tumor status: primary, recurrent	2.32(1.55,3.49)	<0.0001	2.48(1.23,5.01)	0.011	
T category: Ta, T1	1.60(1.05,2.44)	0.029	2.87(1.28,6.41)	0.010	
Carcinoma in Situ: no, yes	1.74(1.00,3.04)	0.052	3.38(1.55,7.40)	0.002	
Grade: G1,G2,G3	1.51(1.10,2.07)	0.010	–	–	
Grade 3: no, yes	–	–	2.38(1.13,5.00)	0.022	

The sections were graded according to the 1973 WHO classification by a pathologist with 10 years’ experience in urology. The pathologist was blinded to the clinical data for all patients. The order of all slices was randomized and regrading of the slices was performed one month later by the same pathologist according to the 2004 WHO classification.The study start time was defined as the time after complete TUR. In all patients, cystoscopies were performed every three months for two years, then every six months to five years and annually thereafter, using a rigid endoscope with 70° optics. The median follow-up duration was 47 months (range, 2–124 months). We define recurrence as development of histologically confirmed urothelial cancer in follow-up after complete resection of NMIBC, and progression as a tumor recurrence with either stage pT2 or higher disease in the bladder invasion [7]. The endpoint for patients without recurrence and progression was the date of the last available follow-up cystoscopy. For patients with recurrence or progression, the endpoint was the time that recurrence/progression confirmed by histopathology.

### Statistical Analysis

Statistical analysis was performed using SPSS version 16.0. The chi-squared test was used to evaluate statistical significance of differences between the data of the two groups; multivariate Cox regression models were used to verify independent predictive parameters of recurrence and progression based on the EORTC risk tables; Kaplan-Meier analysis was used to compare the recurrence-free and progression-free survival according to the 1973 and 2004 WHO classifications, statistical comparison of the data was based on the log-rank test and P-values under 5% were considered statistically significant.

**Figure 1:**
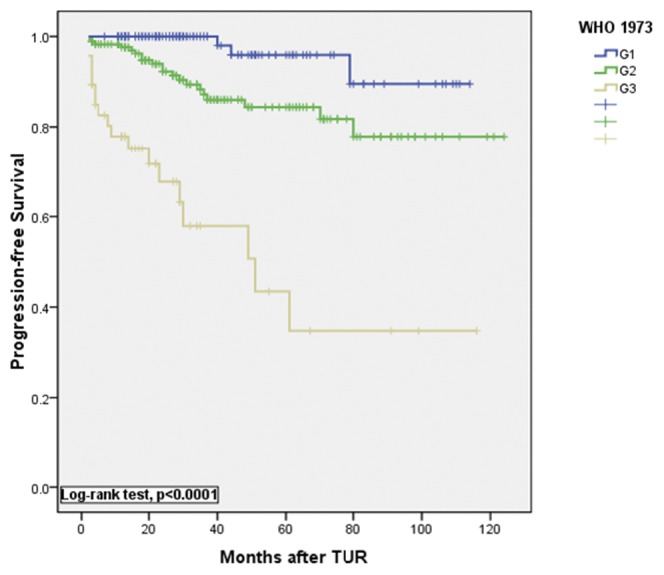
Kaplan-Meier estimates of progression-free survival rates after transurethral resection (TUR) of the bladder tumor according to the 1973 WHO classification.

**Figure 2:**
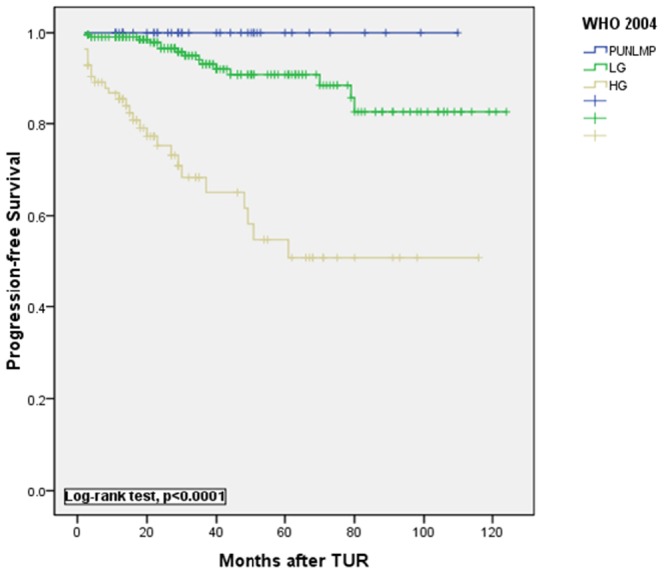
Kaplan-Meier estimates of progression-free survival rates after transurethral resection(TUR) of the bladder tumor according to the 2004 WHO classification.

**Figure 3:**
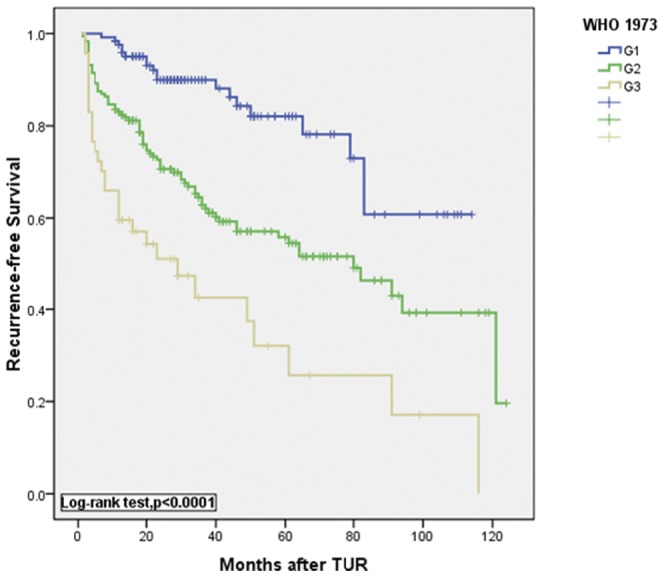
Kaplan-Meier estimates of recurrence-free survival rates after transurethral resection (TUR) of the bladder tumor according to the 1973 WHO classification.

**Figure 4:**
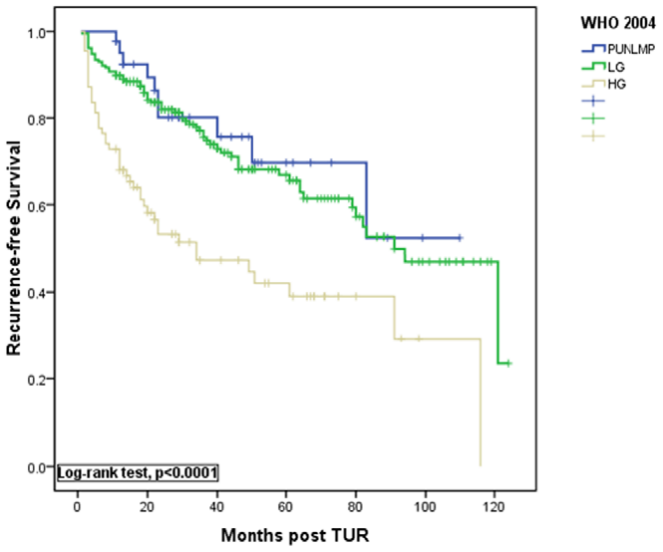
Kaplan-Meier estimates of recurrence-free survival rates after transurethral resection (TUR) of the bladder tumor according to the 2004WHO classification.

## Results

Characteristics of the 348 patients with NMIBC are summarized in Table 1. There are 287 male, and 61female, the ratio of male to female is 4.7∶1, just close to the Western European(23.6 in males and 5.4 in females) [2]. The median age at diagnosis is 68 years (range 21 to 92), median follow-up was 47 months (2–124 months). On follow-up, 122(35.1%) cases showed recurrence and all of them received a completely TUR once more; 41 (11.8%) cases had experienced a progression and almost all of them treated by a radical cystectomy. According to the 1973 WHO classification, 125 (35.9%) patients with NMIBC were graded G1, 176(50.6%) patients were graded G2 and 47 (13.5%) patients were graded G3. During follow-up, the 5-year recurrence-free survival rates corresponding to the three tumor grades were 82.1%, 55.9%, 32.1% respectively and the 5-year progression-free survival rates were 95.9%, 84.4% and 43.3% respectively. The distribution of WHO papillary urothelial neoplasm of PULNMP, LGPUC and HGPUC were 11.5%, 64.1%, and 24.4%, respectively. The corresponding 5-year recurrence-free survival rates were 69.8%, 67.1% and 42.0% respectively and the 5-year progression-free survival rates were 100%, 90.9% and 54.8%, respectively (Table 2).

Multivariate analysis identified that number of tumors, tumor size, prior recurrence rate, CIS, T category and tumor grade in our patients are prognostic variables associated with the risk of tumor recurrence and progression. In our multivariate analysis, the 1973 WHO classification significantly associated with both tumor recurrence and progression (p = 0.010 and p = 0.022, respectively, Talbe3); Talbe4 shows that the 2004 WHO classification correlated with tumor progression (p = 0.019), while was not proved to be a prognostic variable that can predict the risk of tumor recurrence (p = 0.547, Table 4).

**Table 4:** Multivariate analyses of WHO 2004 classification and other clinical parameters inferences to predict tumor recurrence and progression among patients with non-muscle-invasive bladder cancer.

Variable	Recurrence		Progression	
	HR	P value	HR	P value
Number of tumors: Single, multiple	1.96(1.35,2.83)	<0.0001	1.18(0.62,2.25)	0.622
Tumor Size: <3 cm, ≥3 cm	2.11(1.45,3.05)	<0.0001	2.73(1.37,5.45)	0.004
Tumor status: primary, recurrent	2.41(1.62,3.61)	<0.0001	2.61(1.30,5.24)	0.007
T category: Ta, T1	1.87(1.24,2.82)	0.003	2.60(1.13,5.97)	0.025
Carcinoma in Situ: no, yes	1.92(1.08,3.41)	0.026	3.11(1.45,6.69)	0.004
Grade: PUNLMP, LG, HG	1.12(0.78,1.62)	0.547	–	–
HG: no, yes	–	–	2.56(1.17,5.63)	0.019

Kaplan-Meier curves for progression-free survival are shown in Fig. 1 and Fig. 2. Curves showed that both the 1973 WHO and 2004 WHO classifications were significantly associated with progression-free survival (P<0.0001, log-rank test, Fig. 1 and Fig. 2). A significant difference in tumor recurrence was observed among tumor grades G1 to G3 according to the 1973 WHO grading system (p<0.0001, log-rank test, Fig. 3). For the 2004 WHO grading system, although Fig. 4 showed p<0.0001 among PUNLMP, LG and HG, there is a significant overlap was observed between PUNLMP and LG plots (p = 0.616, log-rank test, Fig. 4).

**Figure 5:**
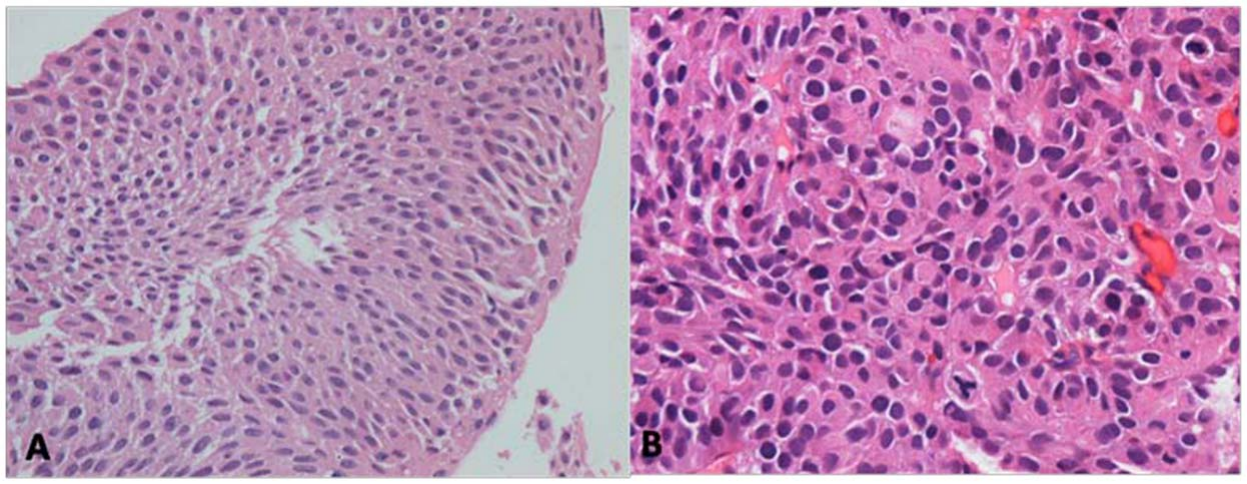
Histologic grading of urothelial tumors: (A) The 2004 WHO classification Papillary urothelial neoplasm of low malignant potential (PUNLMP); (B) the 1973 WHO classification grade 3 urothelial carcinoma.

## Discussion

Despite current research into biomarker identification, histopathological evaluation remains the main method routinely used to determine the prognosis of patients and to aid urologists in the selection of the most appropriate therapy and follow-up stratification. The 1973 WHO grading system has been in widespread use for more than three decades and become well understood by clinicians in predicting clinical behavior of urothelial carcinoma. However, several centers have reported obvious inter-observer variability associated with this system [15], [16], [17]. The new 2004 WHO grading system was developed to avoid the problems clinicians encountered with the WHO 1973 classification and to replace the former system. However, in the study by van Rhijn, the difference of inter-observer variability between the two systems was less marked [18] and there is an ongoing debate about the added value of the 2004 WHO classification versus that of the 1973 system [11], [12], [13]. Therefore, the European Association of Urology (EAU) guidelines advocate the use of both classifications until more prospective trials have been conducted to validate the prognostic value of the WHO 2004 classification [2].

**Figure 6:**
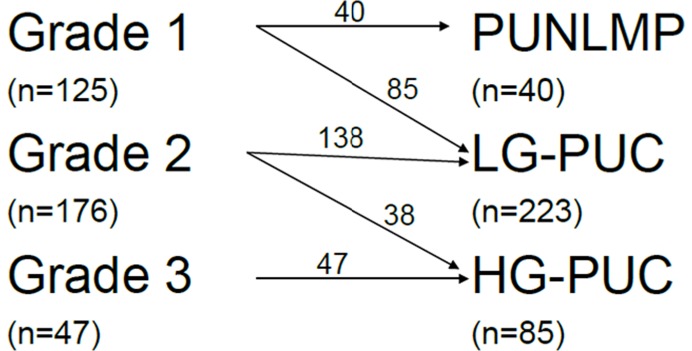
The relationship of the 1973 and 2004 WHO classification in our patients.

In our present study, covering 348 patients with NMIBC, we evaluated the predicting function of both the WHO 1973 and WHO 2004 systems. The WHO 1973 classification is an independent indicator in multivariable analysis for both recurrence and progression in our cohort (Table 3), while the WHO 2004 classification only predicts tumor progression. Furthermore, Kaplan-Meier plots showed a significant overlap between PULNMP and LGPUC in predicting tumor recurrence after completed TUR.

“Papillary urothelial neoplasm of low malignant potential” (PUNLMP, Fig. 5A) is a specific and ground-breaking category introduced by the 2004 WHO classification [19] and is defined as a papillary urothelial tumor that resembles exophytic urothelial papilloma but shows increased cellular proliferation exceeding the thickness of normal urothelium [20]. The introduction of this new category aimed to avoid labeling patients diagnosed with this lesion with the term “cancer” to decrease psychosocial and economic burdens. Several centers reported that these tumors have a significantly lower rate of recurrence and progression than either low- or high-grade UC [21]–[26]. However, these data are not accordance with other studies. Cheng et al. reported that 112 patients with PUNLMP and up to 35 yr of follow-up (median, 12.8years) were at 26.8% risk of local recurrence and 3.6% risk of stage progression [21]. Another study with mean follow-up of 11.7 yr reported a series of 53 patients with PUNLMP, with recurrences in 60%, grade progression to LGPUC in 34% and progression to invasive carcinoma in 8% [27]. Even more, there was report showed that Strong immunohistochemical expression of FGFR3, a superficial staining pattern of CK20, and a low proliferative activity define those papillary urothelial neoplasms of low malignant potential that do not recur [28]. In our retrospective study, 40 PUNLMP cases were identified. During follow-up, no cases of progression were identified, while 8 cases (25%) had a recurrence, including 4 cases as PUNLMP and 6 cases demonstrated grade progression to LGPUC. Therefore, although the risk of progression is quite low for patients with PUNLMP, a proportion of patients experience a recurrence or even grade progression to LGPUC. Furthermore, certain molecular markers have been evaluated such as point mutations in the FGFR3 gene, which were detected in 85% of PUNLMP tumors and in 88% of low-grade carcinomas [29], and in our data, Fig. 4 shows a significant overlap between PULNMP and LGPUC in predicting tumor recurrence after complete TUR. On the other hand, a recommended surveillance protocol which is significantly different from the standard surveillance protocol for LGPUC has not been published for PUNLMP tumors. Therefore, we propose that appropriate clinical follow-up similar to that for patients with LGPUC should be warranted in all patients with PUNLMP.

**Figure 7:**
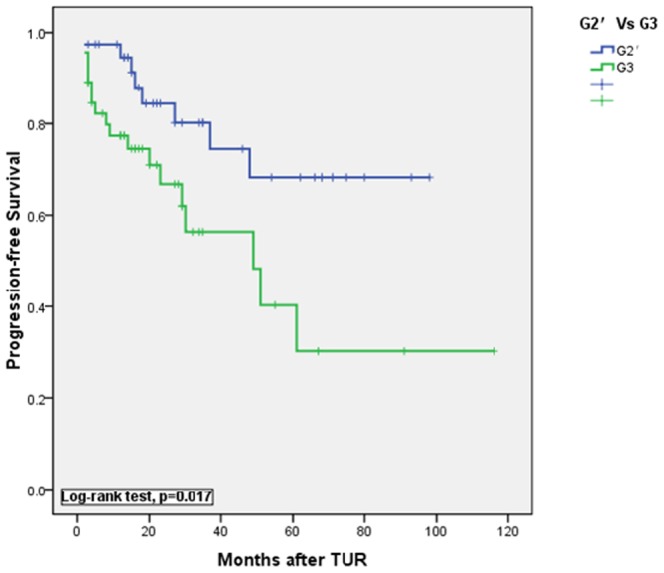
Kaplan-Meier plots of progression-free survival between G3 and G2′(HGPUC but not containing G3 tumors).

The definition for papilloma is the same in both the 1973 WHO and the 2004 WHO classifications [30]. However, this direct translation relationships between the 1973 and the 2004 WHO classifications does not exist. WHO 1973 grade 1 will be either a PULNMP or a LGPUC and grade 2 will be either LGPUC or HGPUC [12]. The similarities and differences between the two grading systems for the 348 cases in this study are shown in Fig. 6. All 40 cases of PUNLMP were graded as G1 tumors and 223 cases of LGPUC included 85 G1 tumors and 138 G2 tumors. All G3 tumors classified according to the 1973 WHO grading system were categorized as HGPUC in accordance with the 2004 WHO classification and 38 (21.6%) of 176 G2 tumors were reclassified as HGPUC (we defined these 38 G2 tumors as G2′ tumors).

This relationship resulted in more frequent diagnosis of HGPUC and more heterogenous groups than that of the former G3 tumors. Furthermore, Kaplan-Meier plots showed significant differences in progression-free survival between the 47 cases of G3 tumors and the 38 cases of G2′ tumors as we had mentioned above (P = 0.017, log-rank test, Fig. 7). The figure shows that G3 tumors are more prone to progression than HGPUC. According to the 1973 WHO classification, G3 demonstrates extreme nuclear abnormalities, disordered architecture, loss of polarity and frequent mitotic activity (Fig. 5B). Therefore, G3 tumors are more aggressive than other classifications and are associated with the poorest prognosis. In this study, 47 G3 urothelial carcinomas were identified by examination of paraffin sections. On follow-up, the 1 yr recurrence-free and progression-free survival was 66.0% and 77.9% respectively and the 5 yr recurrence-free and progression-free survival decreased to 32.1% and 43.4% respectively. With a poor prognosis (especially for tumor progression), a significant difference was observed in G3 as a predictor of recurrence and progression compared with G1 and G2 (Fig. 1 and Fig. 3). Therefore, these data indicate that the 1973 classification system provides a more useful information for determining the cases with more aggressive tumors. Therefore it could enable clinicians in a timely manner to maximize the chances of bladder preservation and cancer control, while minimizing the risks of overtreatment with radical therapy.

So, the 1973 WHO classification is the independent indicator for predicting tumor recurrence and progression; it is a better predictor for determining tumors with more aggressively biological behavior than the 2004 WHO classification.

There are limitations in the present analysis: including retrospective data collection and single cohort study, which may introduce cohort and selection bias, and next, we should make efforts in these areas. Furthermore, all pathology slides were reviewed by a single pathologist, therefore some variability is likely despite extensive experience.

### Conclusions

In conclusion, the results of this analysis have shown that both the 1973 WHO and the 2004 WHO classification predict tumor progression well. However, the former system is superior for predicting tumor recurrence and more useful in predicting cases of aggressive tumors.
